# Heterogeneous Effect of Two Selectin Gene Polymorphisms on Coronary Artery Disease Risk: A Meta-Analysis

**DOI:** 10.1371/journal.pone.0088152

**Published:** 2014-02-03

**Authors:** Zhijun Wu, Yuqing Lou, Lin Lu, Yan Liu, Qiujing Chen, Xin Chen, Wei Jin

**Affiliations:** 1 Department of Cardiology, Ruijin Hospital, Shanghai Jiao Tong University School of Medicine, Shanghai, China; 2 Department of Pulmonary, Shanghai Chest Hospital, Shanghai Jiao Tong University, Shanghai, China; University Medical Center Utrecht, Netherlands

## Abstract

**Background:**

The selectins play important roles in the inflammatory process of coronary artery disease (CAD) and myocardial infarction (MI). Previous studies have shown ambiguous findings regarding a possible association between the selectin genes and CAD. The *E-selectin* Ser128Arg polymorphism and the *P-selectin* Thr715Pro polymorphism have been investigated widely but with inconsistent results. We performed a comprehensive meta-analysis to shed light on this issue.

**Methods:**

Data were extracted by searches of MEDLINE, Embase, CNKI, Wanfang, Google Scholar, PORTA, GeNii, CiNii, J-STAGE, Nurimedia and Koreanstudies Information Service System [Kiss] up to October 2013, in which 10 studies on the Ser128Arg polymorphism with 3369 cases and 2577 controls and 10 studies on the Thr715Pro polymorphism with 5886 cases and 18345 controls. A random-effects model was used to calculate the combined odds ratios. The between-study heterogeneity and publication bias were addressed.

**Results:**

The 128Arg carriers had a significant increased risk of CAD (allele comparison: P = 0.02, OR = 1.33, 95%CI 1.04–1.69, P_heterogeneity_ = 0.01); The 715Pro conferred a non-significant risk reduction relative to the 715Thr (allele comparison: P = 0.40, OR = 0.94, 95%CI 0.82–1.08, P_heterogeneity_ = 0.03).Subgroup analyses demonstrated that the 128Arg carriers had a significant increased risk of CAD among Asians (allele comparison: P = 0.001, OR = 2.07, 95%CI 1.33–3.24, P_heterogeneity_ = 0.77) but not among Caucasians (allele comparison: P = 0.33, OR = 1.13, 95%CI 0.88–1.45, P_heterogeneity_ = 0.08). Carrier status for the 715Pro was significantly associated with reduced risk of MI (allele comparison: P = 0.04, OR =  0.81, 95%CI 0.67–0.99, P_heterogeneity_ = 0.14). The asymmetric funnel plot and the Egger's test (P = 0.041) suggested the presence of publication bias for the Ser128Arg polymorphism.

**Conclusion:**

Our results suggested there is an increase in the risk of CAD conferred by the Ser128Arg polymorphism and the thr715Pro polymorphism may be a protective factor of MI.

## Introduction

It has been known for years that coronary artery disease (CAD) and myocardial infarction (MI) are the main killers in the world. The pathogenesis of CAD and MI involves important inflammatory components [1], [2]. One of the critical steps of inflammatory processes is the migration of leukocytes from the vessel wall to atherosclerosis lesion[3], which is strictly regulated by a variety of cell adhesion molecules and leukocyte-activating factors[4]. Leukocytes is captured from the blood stream and then rolled along the endothelial cell surface. The process is mediated by the selectins, a special family of three cell adhesion molecules: P-selectin, E-selectin and L-selectin. The three selectins have fundamental differences in their distribution, activation and expression. E-selectin is only expressed on the activated endothelium and recruits polymorphonuclear leukocytes, myeloid cells and T-lymphocytes to the inflammation sites[5]; P-selectin is expressed on the endothelial cells as well as platelets and instantly migrates to the plasma membrane when exposed to some free radicals, nitric oxide synthase inhibitors and other mediators[6]; L selectin is expressed on most types of leukocytes, as well as some neutrophils and myeloid cells [5] and serves as a homing receptor for lymphocytes to peripheral lymph nodes. L-selectin recruits cells to inflammation lesion like E-selectin.

Although the in vivo study has suggested that E-selectin and P-selectin exert similar effects of leukocyte adhesion on atherosclerosis lesions. The exact roles of E-selectin and P-selectin remain unclear. Estimates have placed genetic predisposition to be responsible for 40–60% of susceptibility to CAD [2], [7]–[9]. The single nuclear polymorphisms (SNPs) of adhesion molecules act independently of known risk factors of severe atherosclerosis [10]. Several polymorphisms of the selectin genes have been mentioned to contribute to the CAD incidence. For instance, an adenine to cytosine substitution at cDNA position 561, resulting in an amino acid exchange from serine (Ser) to arginine (Arg), in the epidermal growth factor (EGF) -like domain of E-selectin has profound effects on atherosclerosis. In additional, a Thr715Pro polymorphism located in front of the transmembrane domain of the *P-selectin* gene is associated with the susceptibility to CAD and MI. These two polymorphisms were the most investigated, but with conflicting results. It should be considered that the sample size in the previous studies was relatively small. Hence, the studies had low statistical power to detect modest associations of genotype with CAD. To shed some light on this divergence and maximize statistical power, we sought to evaluate the correlation of these two polymorphisms of the selectin genes with CAD by performing a comprehensive meta-analysis of available individual studies. Furthermore, the between-study heterogeneity and the publication bias were addressed.

## Materials and Methods

### Data Source and Search Strategy

We followed the guidelines of the 2009 preferred reporting items for systematic reviews and meta-analyses (PRISMA) statement [11] when performed the literature search (Checklist S1). The search strategy was conducted by searching from PubMed/MEDLINE, Embase, China Nation Knowledge Infrastructure (CNKI) Platform and Wanfang electronic databases up to October 2013. In addition, five Japanese electronic databases (PORTA, GeNii, CiNii, J-STAGE and Google Scholar [Japanese]) as well as three Korean electronic databases (Nurimedia, Koreanstudies Information Service System [Kiss] and Google Scholar [Korean]) were also retrieved. The following medical subject heading (MeSH) terms and key words were used in the computer-based searches: ‘Ser128Arg’ or ‘A561C’, ‘E-selectin’ or ‘SELE’, ‘Thr715Pro’ or ‘rs6136’, ‘P-selectin’ or ‘SELP’ and ‘coronary artery disease’ or ‘acute coronary syndrome’ or ‘myocardial infarction’ or ‘atherosclerosis ’. We scanned the titles and abstracts of potentially appropriate articles followed by determining whether these studies conformed to our inclusion criteria. If the suitability of a study was uncertain, we scrutinized the full text for further estimation. We identified additional studies by screening the bibliographies of relevant studies and reviews. The related search function of Pubmed was also used.

### Selection Criteria

All the studies conformed to the following criteria were potentially involved: (1) Studies followed case-control or cross-sectional design (studies without controls were excluded); (3) Studies providing adequate allele or genotype information on the examined polymorphisms; (4) Studies published in English journals or their supplements; (5) If multiple studies derived from a common population, only the study with the largest sample size was extracted to avoid overlapping data; (6) The genotype frequency amongst control must conform to Hardy-Weinberg equilibrium (HWE).

Standard definitions were used to diagnosis disease outcomes [12].CAD was defined as significant stenosis with ≥50% luminal obstruction in at least one or more major coronary vessel [13]. Acute coronary syndrome (ACS) was defined by unstable angina pectoris and fatal or non-fatal MI [14] and MI was defined by the World Health Organization (WHO) criteria [15].

### Data Extraction

Two authors (Z.W. and Y.Lou.) independently extracted the relevant information from qualified studies by the standard data-collection protocol according to the inclusion criteria. The data were entered into separate databases in duplicate by the two authors. Any encountered inconsistencies were resolved at a consensus meeting. The following data was gathered based on different cohort: first author's name, publication year, ethnicity, geographic location, study design, population source, disease outcomes, matching variables, clinical characteristics (such as gender, age, body mass index [BMI], the percentage of hypertension [HTN], diabetes mellitus [DM], smoking status), the distribution of the Ser128Arg and Thr715Pro genotype in both patients and controls and conformity to genotype frequencies with HWE. Continuous variables were manifested as mean ± standard deviation (SD) or median (5th and 95th percentiles).

### Statistical Analysis

Data management and processing were performed using Review Manager software release 5.0 (Oxford, England) and Stata 11.0 (Stata Corporation, College Station, Texas, USA). The combined odd ratios (ORs) corresponding to 95% confidence intervals (CIs) were calculated under the random-effects model using DerSimonian & Laird method. Considering the relatively low number of homozygote genotype, we only estimate the effect size of these two polymorphisms on CAD under the allelic and dominant models. The possibility of heterogeneity was assessed using Cochran's chi-square based Q statistic test [16] and index I^2^ statistic. A significant Q statistic test (P<0.10) indicated heterogeneity among studies. I^2^ = 100%×(Q−df)/Q, measures the proportion of the between-study variability due to heterogeneity rather than chance[17], [18]. Departure from HWE was examined via the chi-square test or Fisher's exact test on the basis of a Web program (http://ihg2.helmholtz-muenchen.de/cgi-bin/hw/hwa1.pl).

A univariate meta-regression was used to explore potential factors associated with the genetic heterogeneity. A cumulative meta-analysis might indicate the influence of the initial published research on subsequent studies and the evolution of the combined estimates with ascending date of publication. A sensitivity analysis was conducted using one-study removal approach to identify the influence of each study on the overall effect size [19]. The possibility of publication bias could be visual by an funnel plot [20] and then assessed by the Egger's and the Begg-Mazumdar test as well as the trim-and-fill method. All P values were 2-sided. P<0.05 was considered significant except the Q statistic test.

## Result

### Literature Search

**Figure 1:**
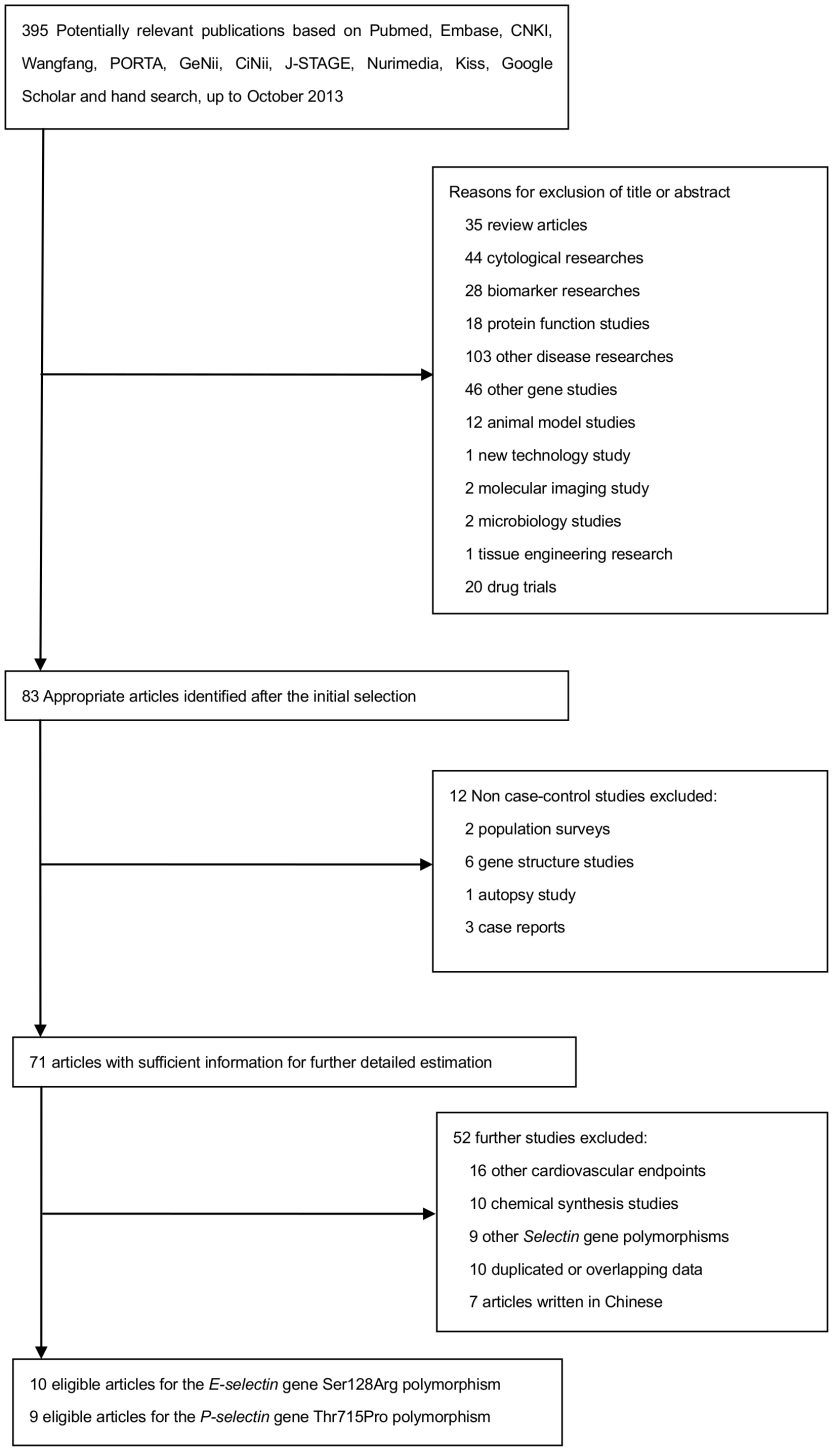
Flowchart diagram illustrating search strategy and study selection.

A total of 395 relevant citations were reviewed based on our selection strategy. The stepwise selection process is shown in Figure 1. After sequent selection, a total of 19 articles (10 of the *E-selectin* Ser128Arg polymorphism and 9 of the *P-selectin* Thr715Pro polymorphism) with adequate information satisfied our inclusion criteria. The study by Morgan *et al.* [21] provided the genotype information of both the two polymorphism and regarded ACS as the disease endpoint. Because the population of the study by Volcik *et al.* [22] derived from two different ethnic backgrounds (Caucasian & African), we considered them as two independent studies and calculated the ORs separately. The Indian population of the study by Tripathi *et al.* [23] was regarded as an independent group because its lineage is miscellaneous and cannot simply be grouped as Asian or Caucasian [24]–[26]. In general, 15 studies were performed among Caucasians [21], [22], [27]–[39] and 2 among Asians [40], [41]. Most of the studies used a retrospective case-control design [21], [23], [27]–[34], [36]–[41] except 2 studies[22], [35].P-B controls were adopted in 8 studies[22], [27], [28], [30], [33]–[35], [37] and H-B in 10 studies[21], [23], [31], [32], [36], [38]–[42]. Cases and controls were matched in 8 studies [21], [23], [30], [32]–[35], [37]. CAD was employed as the disease endpoint in 12 of the studies [22], [23], [27]–[32], [37]–[39], [41], while MI was taken as the outcome in 5 studies [33]–[36], [40].

### Data Synthesis

There were 10 studies with 3,369 CAD patients and 2,577 controls for the *E-selectin* Ser128Arg polymorphism and 10 studies with 5,886 cases and 18,345 controls for the *P-selectin* Thr715Pro polymorphism involved in our meta-analysis. The clinical characteristics of included studies are shown in Table 1. The allele and genotype frequencies of these two polymorphisms are listed in Table 2 and 3 respectively. All the studies conformed to HWE (P_HWE_>0.05).

**Table 1:** Summary of all included studies in the meta-analysis.

First Author	Year	Ethnicity	Geographic location	Design	Source	Endpoints	Matched	Status	Age,year	Gender,M(%)	HTN,%	DM,%	Smoke,%	BMI,kg/m^2^
Abu-Amero KK	2007	Caucasian	Saudi	Retrospective	H-B	CAD	no	cases	54.2±11.9	69.0	83.7	91.6	30.9	–^a^
								controls	55.7±11.8	55.7	62.8	67.2	44.7	–
Barbaux SC	2001	Caucasian	Germany	Retrospective	P-B	CAD	no	cases	62.3±10.2	74.1	–	–	23.9	26.9±3.7
								controls	61.3±7.5	70.4	–	–	13.5	26.6±5.0
Bugert P	2004	Caucasian	Germany	Retrospective	P-B	CAD	no	cases	59.5±16.9	74.3	52.1	13.8	34.8	–
								controls	54.4±17.1	51.4	0.0	–	–	–
Carter AM	2003	Caucasian	UK	Retrospective	P-B	CAD	yes	cases	59(52–65)	65.0	32.0	8.0	74.0	26.7(26.2–27.1)
								controls	63(49–67)	56.0	15.0	1.0	41.0	25.5(25.0–25.9)
Endler G	2003	Caucasian	Austria	Retrospective	H-B	CAD	no	cases	64(57–74)	69.2	62.7	100.0	37.3	27.6(25.0–29.8)
								controls	64(56–75)	56.5	50.7	100.0	15.9	27.7(23.6–30.9)
Ghazouani L	2011	Caucasian	Tunisia	Retrospective	H-B	CAD	yes	cases	58.2±11.7	78.2	38.1	52.7	41.4	27.3±4.3
								controls	56.5±14.5	76.1	12.3	9.1	21.5	25.3±2.4
Herrmann SM	1998	Caucasian	France&UK	Retrospective	P-B	MI	yes	cases	54.0±8.2	100.0	–	–	–	–
								controls	53.0±8.5	100.0	–	–	–	–
Kee F	2000	Caucasian	UK	Retrospective	P-B	MI	yes	cases	57.2	56.2	–	–	79.3	27.5
								controls	58.1	55.0	–	–	61.4	26.8
Li Y	2002	Asian	China	Retrospective	H-B	CAD	no	cases	62.1±10.6	67.7	–	–	54.4	24.8±3.1
								controls	60.5±11.7	66.8	–	–	29.7	24.0±3.6
Morgan TM	2007	Caucasian	US	Retrospective	H-B	ACS	yes	cases	60.7±12.5(M)	67.8	60.1	23.8	33.1	29.1±5.5(M)
									63.1±13.2(F)					29.9±6.9(F)
								controls	60.0±12.1(M)	60.6	51.2	11.9	13.2	27.9±5.0(M)
									61.8±12.8(F)					27.7±6.9(F)
Rosner SA	2005	Caucasian	US	Prospective	P-B	MI	yes	cases	58.7±8.6	100.0	25.1	5.6	15.1	25.5±3.3
								controls	58.8±8.5	100.0	15.2	2.7	15.3	25.0±3.0
Sakowicz A	2009	Caucasian	Poland	Retrospective	H-B	MI	no	cases	41.0±4.9	83.4	38.0	0.0	87.1	–
								controls	54.0±8.6	47.1	15.0	0.0	58.6	–
Tripathi R	2009	Indian	India	Retrospective	H-B	CAD	yes	cases	57.1±9.7	79.0	47.4	43.2	20.1	25.5±3.0
								controls	53.2±9.2	74.0	23.0	24.8	10.0	25.1±3.1
Volcik KA	2006	Caucasian	US	Prospective	P-B	CAD	no	cases	55.6±3.9	66.0	47.0	24.0	34.0	28.2±3.9
		& African						controls	53.8±5.7	41.0	32.0	9.0	25.0	27.6±5.7
Wenzel K	1997	Caucasian	German	Retrospective	P-B	CAD	yes	cases	42(25–50)	87.6	46.9	6.2	89.4	–
								controls	38	90.3	–	–	–	–
Ye SQ	1999	Caucasian	US	Retrospective	H-B	CAD	no	cases	–	57.3	–	–	–	–
								controls	–	45.1	–	–	–	–
Yoshida M	2003	Asian	Japan	Retrospective	H-B	MI	no	cases	57.7±8.1	76.3	–	–	–	–
								controls	47.6±7.9	70.0	–	–	–	–
Zak I	2008	Caucasian	Poland	Retrospective	H-B	CAD	no	cases	43.8±6.1	66.5	–	–	55.5	26.8±4.3
								controls	35.3±10.5	75.9	–	–	23.2	24.8±3.7

P-B:population-based study; H-B:hospital-based study; CAD: coronary artery disease; MI: myocardial infarction; ACS: acute coronary syndrome; M(%):male(percent); F:female; HTN: hypertension; DM: diabetes mellitus; BMI: body mass index; ^a^:data not available; Age and BMI are expressed as mean ±SD (standard deviation) or median (5th and 95th percentiles)

**Table 2:** The frequencies of the *E-selectin* Ser128Arg polymorphism among CAD and controls.

First Author	Sample size		Arg allele,%		Ser allele,%		ArgArg genotype		SerArg genotype		SerSer genotype		HWE
	cases	controls	cases	controls	cases	controls	cases	controls	cases	controls	cases	controls	P value
Abu-Amero KK	1112	427	6.4	6.4	93.6	93.6	–^a^	–	–	–	–	–	
Endler G	185	69	11.4	13.0	88.6	87.0	3	0	36	18	146	51	0.21
Li Y	248	256	6.7	3.1	93.3	96.9	0	0	33	16	215	240	0.61
Morgan TM	811	650	9.4	10.3	90.6	89.7	7	6	137	123	658	528	0.69
Sakowicz A	163	140	–	14.3	–	85.7	–	5	–	29	116	102	0.12
Tripathi R	329	331	8.5	5.6	91.5	94.4	0	0	56	37	273	294	0.28
Wenzel K	113	103	15.5	8.7	84.5	91.3	1	2	33	14	79	87	0.13
Ye SQ	82	71	19.5	10.6	80.5	89.4	2	0	28	15	52	56	0.32
Yoshida M	135	327	6.3	3.4	93.7	96.6	0	0	17	22	118	305	0.53
Zak I	191	203	12.0	10.8	88.0	89.2	4	2	38	40	149	161	0.78
Total	3369	2577	8.7	7.7	91.3	92.3	17	15	378	314	1806	1824	

HWE: Hardy–Weinberg equilibrium. The P-value of HWE determined by the χ^2^ test or Fisher's exact test among controls; ^a^:data not available.

**Table 3:** The frequencies of the *P-selectin* Thr715Pro polymorphism among CAD and controls.

First Author	Sample size		Pro allele,%		Thr allele,%		ProPro genotype		ThrPro genotype		ThrThr genotype		HWE
	cases	controls	cases	controls	cases	controls	cases	controls	cases	controls	cases	controls	P value
Barbaux SC	869	334	–^a^	–	–	–	–	–	–	–	634	255	
Bugert P	261	214	13.2	9.6	86.8	90.4	7	3	55	35	199	176	0.41
Carter AM	249	252	10.3	10.4	89.7	89.6	3	2	45	48	199	201	0.64
Ghazouani L	298	339	2.5	4.1	97.5	95.9	0	0	17	24	319	267	0.46
Herrmann SM	647	758	8.9	12.5	91.1	87.5	4	14	99	136	501	507	0.18
Kee F	696	647	11.7	14.1	88.3	85.9	8	10	138	155	512	455	0.44
Morgan TM	811	650	10.4	9.8	89.6	90.2	9	2	150	124	646	530	0.06
Rosner SA	522	2089	9.8	10.1	90.2	89.9	3	21	96	380	423	1688	0.94
Volcik KA [African]	337	3657	2.3	2.2	97.7	97.8	0	1	14	138	296	3043	0.66
Volcik KA [Caucasian]	1196	9405	11.5	11.1	88.5	88.9	16	113	224	1802	876	7243	0.94
Total	5886	18345	9.7	9.3	90.4	90.7	50	166	838	2842	4605	14365	

HWE: Hardy–Weinberg equilibrium. The P-value of HWE determined by the χ^2^ test or Fisher's exact test among controls; ^a^:data not available.

The overall frequency of the *E-selecitn* 128Arg allele was 8.7% in cases and 7.7% in controls. The frequency of the 128Arg allele among Caucasians (9.0% cases & 9.0% controls) was moderately higher than that among Asians (6.5% cases & 3.3% controls). As for the *P-selectin* Thr715Pro polymorphism, most of the qualified studies were performed among Caucasians except one study (Volcik *et al.*[African] [22]). The 715Pro allele proportion in cases (9.7%) was similar to that in controls (9.3%).

**Figure 2:**
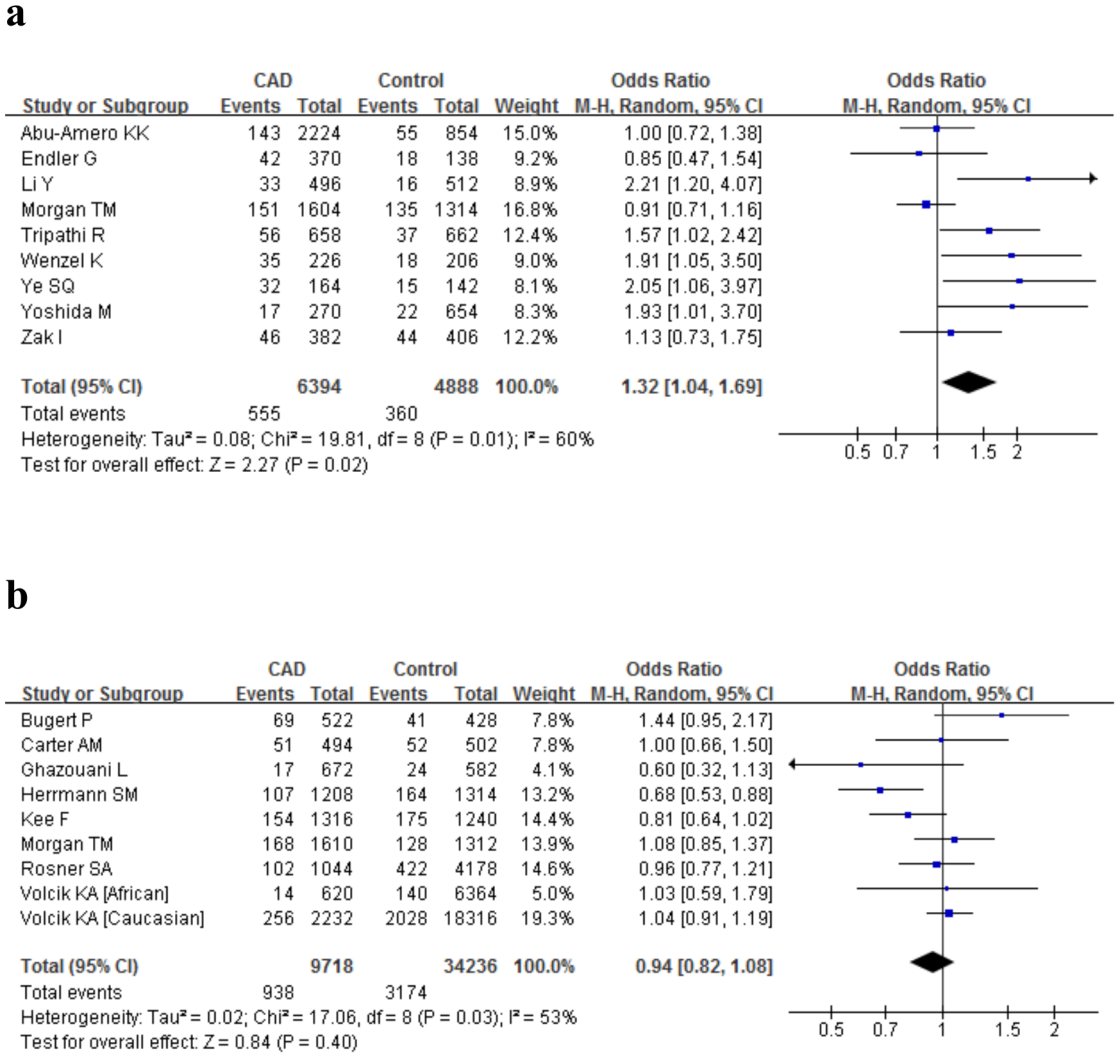
Forest plots detailing the association of the *E-selectin* Ser128Arg polymorphism (Arg versus Ser, Figure 2a) and the *P-selectin* Thr715Pro polymorphism (Pro versus Thr, Figure 2b) with CAD using the allelic model in overall analysis.

Table 4 and 5 summarized the estimates of genotype data for allelic and dominant models in total and subgroup analysis. In the main analyses, the overall comparison of the *E-selectin* 128Arg allele relative to 128Ser yielded a significantly increased risk of CAD (allele comparison: P = 0.02, OR = 1.33, 95%CI 1.04–1.69, P_heterogeneity_ = 0.01; dominant model: P = 0.02, OR = 1.40, 95%CI 1.06–1.87, P_heterogeneity_ = 0.02) with remarkable heterogeneity(Figure 2a); No predominant association was observed for the *P-selectin* Thr715Pro polymorphism with CAD under the both allelic (P = 0.40, OR = 0.94, 95%CI 0.82–1.08, P_heterogeneity_ = 0.03) and dominant model(P = 0.22, OR = 0.92, 95%CI 0.82–1.05, P_heterogeneity_ = 0.10) (Figure 2b).

**Table 4:** Summary estimates of the *E-selectin* Ser128Arg polymorphism with the CAD risk under the allelic and dominant models.

study population	study number,	Allele comparison (Arg versus Ser)					Dominant model(ArgArg+SerArg versus SerSer)				
	(case/control),n(n/n)	P value^a^	OR	95% CI	I^2^,%	P_heterogeneity_	P value	OR	95% CI	I^2^,%	P_heterogeneity_
*Total studies*	10(3369/2577)	0.02	1.33	1.04–1.69	59.6	0.01	0.02	1.40	1.06–1.87	62.5	0.02
											
*Ethnicity*											
Asian	2(383/583)	0.00	2.07	1.33–3.24	0.0	0.77	0.00	2.16	1.37–3.40	0.0	0.76
Caucasian	7(2657/1663)	0.33	1.13	0.88–1.45	49.0	0.08	0.27	1.20	0.87–1.66	57.9	0.04
											
*Matched*											
Yes	3(1253/1084)	0.25	1.33	0.82–2.15	75.8	0.02	0.05	1.42	0.80–2.51	49.3	0.08
No	7(2116/1493)	0.06	1.35	0.98–1.85	54.3	0.05	0.23	1.42	1.00–1.99	80.3	0.01
											
*Disease endpoint*											
CAD	7(2260/1460)	0.02	1.38	1.06–1.80	50.1	0.06	0.02	1.54	1.09–2.19	53.4	0.02
MI	2(298/467)	0.05	1.93	1.01–3.70	–^a^	–	0.13	1.47	0.89–1.43	31.0	0.13
											
*Publication Date*											
<2007	5(763/826)	0.00	1.68	1.18–2.40	38.8	0.16	0.00	1.68	1.18–2.40	38.8	0.16
≥2007	5(2606/1751)	0.53	1.07	0.86–1.34	39.0	0.18	0.53	1.07	0.86–1.34	39.0	0.18

a:Test for overall effect; CAD: coronary artery disease; MI: myocardial infarction; ^a^:data not available.

**Table 5:** Summary estimates of the *P-selectin* Thr715Pro polymorphism with the CAD risk under the allelic and dominant models.

study population	study number,	Allele comparison (Pro versus Thr)					Dominant model(ProPro+ThrPro versus ThrThr)				
	(case/control),n(n/n)	P value^a^	OR	95% CI	I^2^,%	P_heterogeneity_	P value	OR	95% CI	I^2^,%	P_heterogeneity_
*Total studies*	10(5886/18345)	0.40	0.94	0.82–1.08	53.1	0.03	0.22	0.92	0.82–1.05	39.3	0.10
											
*Design*											
Prospective	3(2055/15151)	0.74	1.02	0.91–1.14	0.0	0.85	0.73	1.02	0.90–1.16	0.0	0.94
Retrospective	7(3831/3194)	0.38	0.91	0.72–1.13	64.9	0.01	0.14	0.88	0.73–1.05	47.0	0.08
											
*Source*											
P-B	8(4777/17356)	0.46	0.94	0.80–1.10	57.3	0.03	0.27	0.93	0.80–1.06	43.2	0.09
H-B	2(1109/989)	0.62	0.87	0.50–1.51	64.6	0.09	0.54	0.85	0.50–1.43	59.8	0.12
											
*Matched*											
Yes	6(3223/4735)	0.09	0.87	0.74–1.02	46.5	0.10	0.06	0.86	0.74–1.01	32.3	0.19
No	4(2663/13610)	0.30	1.09	0.93–1.27	8.0	0.34	0.77	1.03	0.86–1.23	24.9	0.26
											
*disease endpoint*											
CAD	6(3210/14201)	0.65	1.04	0.87–1.25	24.5	0.26	0.87	0.99	0.83–1.17	25.6	0.24
MI	3(1865/3494)	0.04	0.81	0.67–0.99	49.2	0.14	0.05	0.82	0.67–1.00	44.6	0.16
											
*Publication Date*											
<2005	5(2722/2205)	0.50	0.91	0.68–1.21	69.8	0.02	0.19	0.87	0.70–1.07	49.7	0.09
≥2005	5(3164/16140)	0.77	1.02	0.92–1.13	0.0	0.53	0.89	1.01	0.90–1.13	0.0	0.59

a :Test for overall effect; P-B:population-based; H-B:hospital-based; CAD: coronary artery disease; MI: myocardial infarction.

### Sensitivity Analysis

Sensitivity analyses demonstrated that no single study influenced the heterogeneity and ORs when evaluating the association between the *E-selectin* Ser128Arg polymorphism and CAD in total analysis. As for the *P-selectin* Thr715Pro polymorphism, the study by Herrmann *et al*.[33] contributed to the observed heterogeneity in total analysis. The overall heterogeneity no longer existed (P_heterogeneity_  = 0.21) after removing this study and the effect size did not change materially.

### Cumulative Analysis and Publication Bias

**Figure 3:**
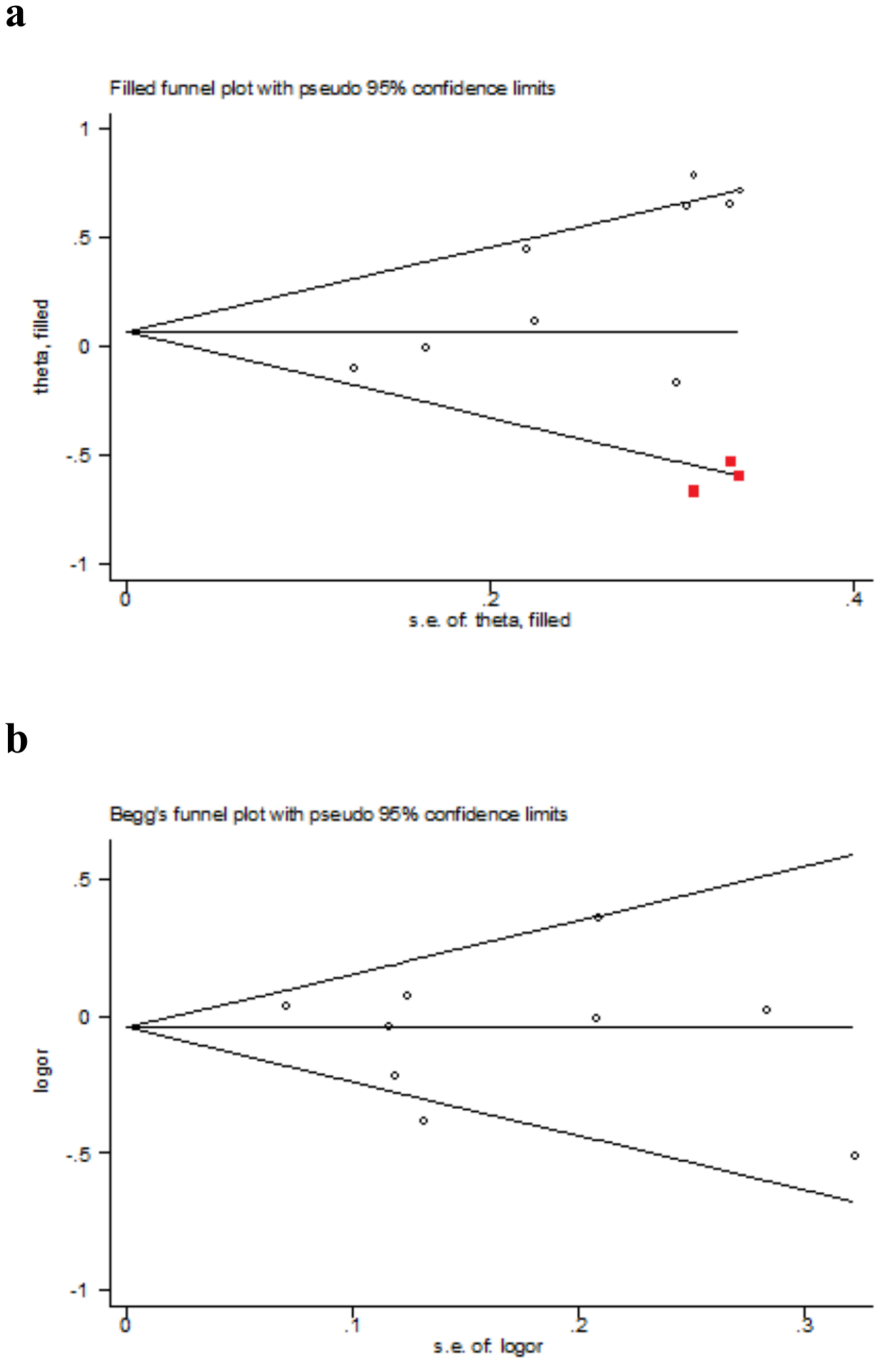
Filled Begg's funnel plot for studies investigating the effect of the *E-selectin* Ser128Arg polymorphism on CAD (Figure 3a) and Begg's funnel plot analysis of the *P-selectin* Thr715Pro polymorphism with CAD risk (Figure 3b). Red squares are missed studies due to publication bias in Figure 3a.

Cumulative trends for allelic contrast of these two polymorphisms suggested there was not any distinct evidence for the first published study that triggered the ensuing replication of other studies that tried to replicate this initial result (data not shown).The funnel plot for the Ser128Arg polymorphism was distinct asymmetric for small negative studies. The Egger's (P = 0.041) and the Begg-Mazumdar (P = 0.016) test suggested a presence of publication bias. By using the trim and fill method, we found that if the publication bias was the sole source of the funnel plot asymmetry, 3 studies were needed to be symmetrical (Figure 3a). For the Thr715Pro polymorphism, the funnel plot analysis as well as the Egger's and the Begg-Mazumdar test reflected a low probability of publication bias under the allele comparison (P = 0.91 for Egger's test and P = 0.92 for Begg-Mazumdar test) (Figure 3b).

### Subgroup Analysis

**Figure 4:**
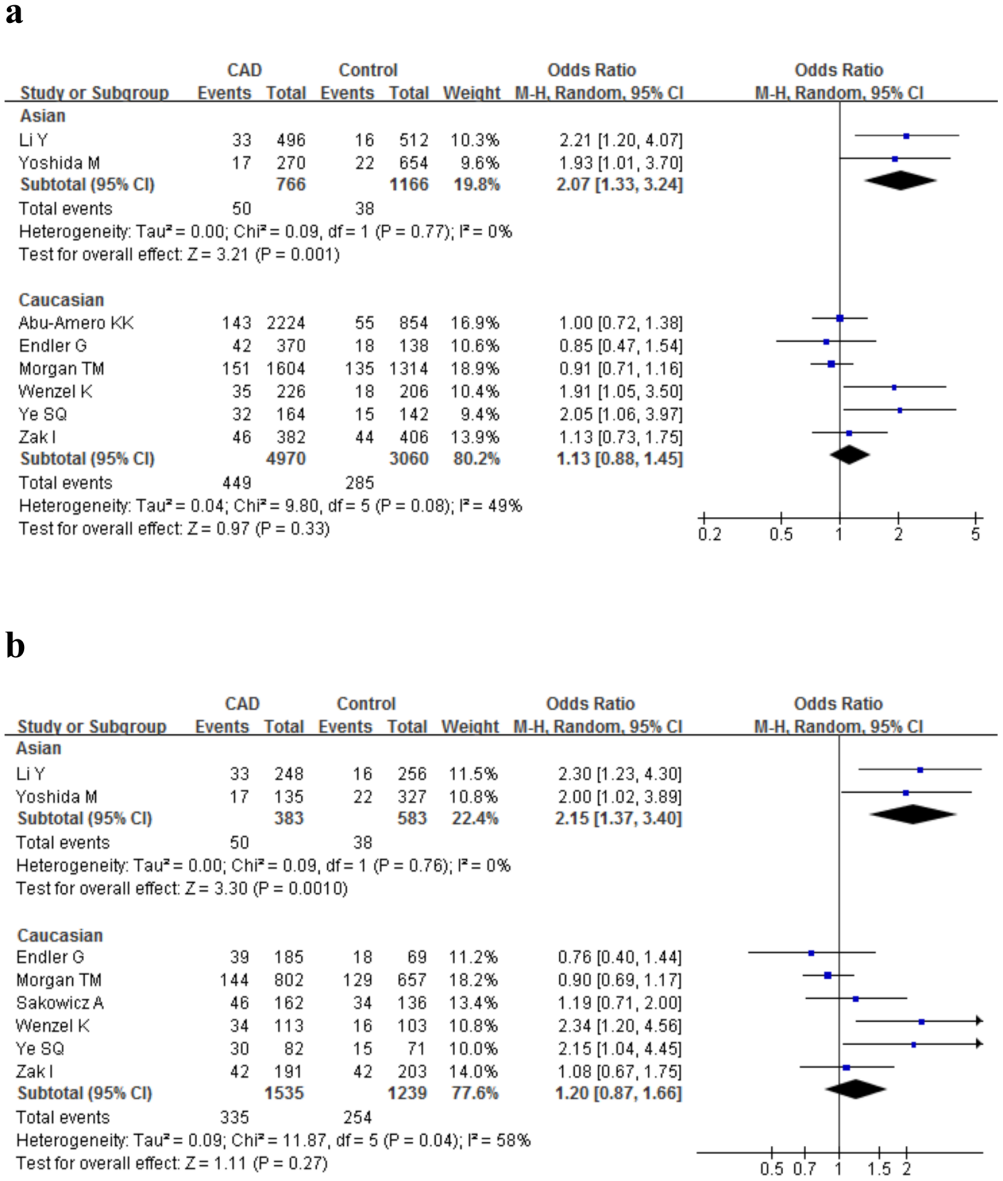
ORs and 95%CI of individual studies and pooled data for the association between the *E-selectin* Ser128Arg polymorphism and CAD among Asians under the allele comparison (Arg versus Ser, Figure 4a) and under the dominant model (ArgArg+SerArg versus SerSer, Figure 4b).

To evaluate the possible factors that contributed to the majority of heterogeneity, we undertook a set of subgroup analyses. ORs generated for the Ser128Arg polymorphism were statistically significant among Asians (allele comparison: P = 0.001, OR = 2.07, 95%CI 1.33–3.24, P_heterogeneity_ = 0.77) but not among Caucasians (allele comparison: P = 0.33, OR = 1.13, 95%CI 0.88–1.45, P_heterogeneity_ = 0.08) (Figure 4); When data were stratified according to disease type, there was a significant association of the Ser128Arg polymorphism with CAD observed (allele comparison: P = 0.02, OR = 1.38, 95%CI 1.06–1.80, P_heterogeneity_ = 0.06; dominant model: P = 0.02, OR = 1.54, 95%CI 1.09–2.19, P_heterogeneity_ = 0.02); The 128Arg allele carriers had a marginal risk increase (42%) of CAD in matched studies under the dominant model (P = 0.05, 95%CI 0.80–2.51, P_heterogeneity_ = 0.08). Data were then stratified according to publication year. The out points of the publication year (2007 for the *E-selectin* Ser128Arg polymorphism & 2005 for the *P-selectin* Thr715Pro polymorphism) were chosen as the median. Risk increase in the studies published before 2007 (allele comparison: P = 0.004, OR = 1.68, 95%CI 1.18–2.40, P_heterogeneity_ = 0.16) was significantly different from that in the studies published in/after 2007(allele comparison: P = 0.53, OR = 1.07, 95%CI 0.86–1.34, P_heterogeneity_ = 0.18) (Table 4).

**Figure 5:**
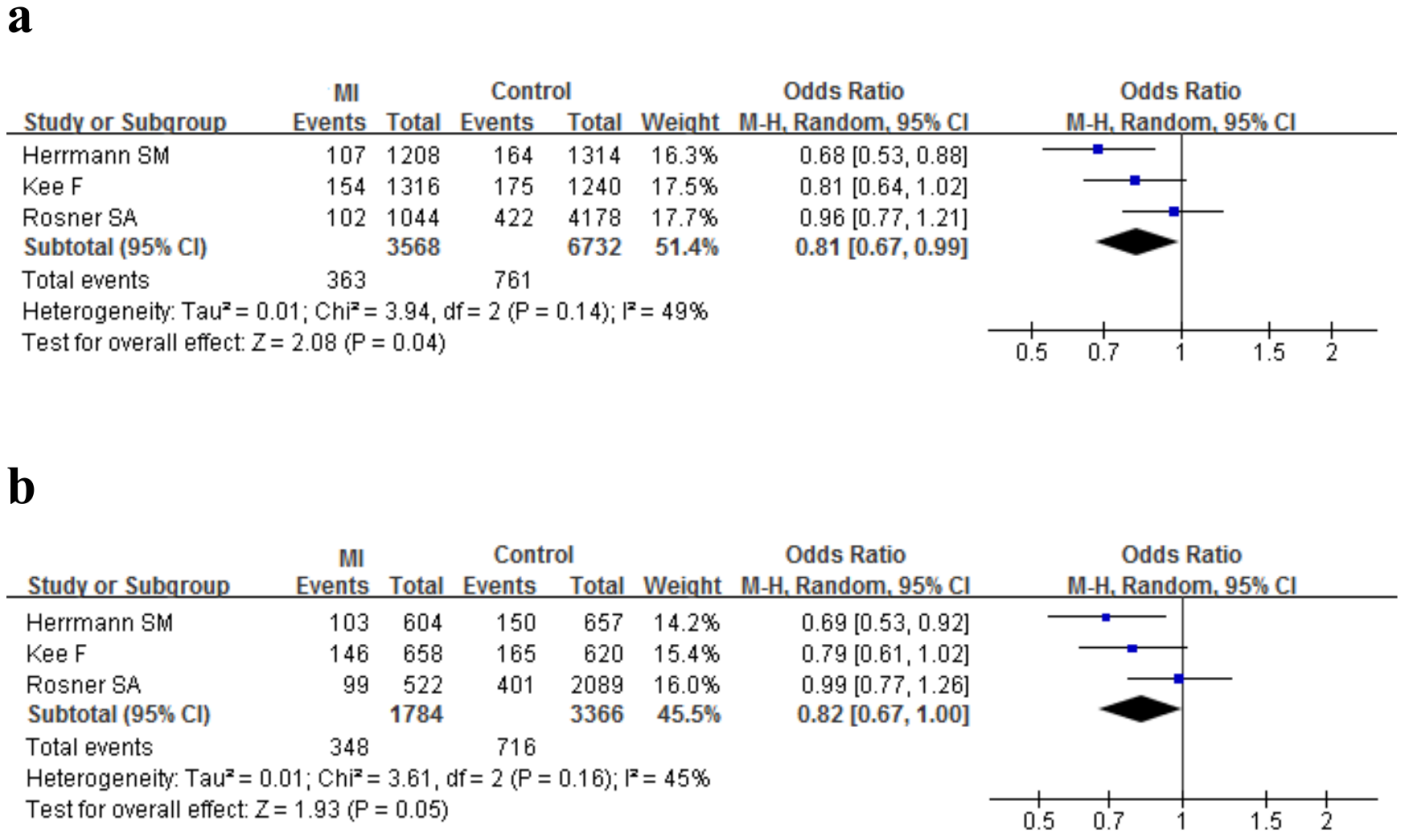
Meta-analysis for the association between the *P-selectin* Thr715Pro polymorphism and MI under the allele comparison (Pro versus Thr, Figure 5a) and under the dominant model (ProPro+ThrPro versus ThrThr, Figure 5b).

As for the *P-selectin* Thr715Pro polymorphism, the 715Pro carriers had a significant 19% risk reduction in MI group (allele comparison: P = 0.04, 95%CI 0.67–0.99, P_heterogeneity_ = 0.14) and a suggestive risk effect in CAD group (allele comparison: P = 0.65, OR = 1.04, 95%CI 0.87–1.25, P_heterogeneity_ = 0.26) (Figure 5). In addition, we observed a marginal association of the Thr715Pro polymorphism with CAD in matched studies (allele comparison: P = 0.09, OR = 0.87, 95%CI 0.74–1.02, P_heterogeneity_ = 0.10; dominant model: P = 0.06, OR = 0.86, 95%CI 0.74–1.01, P_heterogeneity_ = 0.19). However, there is a lack of dramatic association of the Thr715Pro polymorphism with CAD risk when data were categorized according to study design (prospective/retrospective), population source (P-B/H-B) and publication date (<2005/≥2005)(Table 5).

### Meta-regression Analysis

A univariate meta-regression analysis was used to detect the effect of potential factors on genetic heterogeneity. Single covariates such as ethnicity, population source, case definition, study design, matching information, language, publication year and the baseline characteristics of total population were added in a series of univariate models. There was no evidence suggesting these factors were responsible for the between-study heterogeneity for both the *E-selectin* Ser128Arg polymorphism and the *P-selectin* Thr715Pro polymorphism.

## Discussion

There has been growing interest in the role of cellular adhesion molecules in atherosclerosis. Among these, the selectins play essential roles in the first step in leukocyte recruitment and infiltration, which is vital in the key process of atherosclerosis[3]. The association between the SNPs of the selectin genes with CAD was previously confounding because of contradictory results produced by a range of relatively small individual studies. In addition, true variability across different population[43] and different genetic backgrounds[44] may be the other explanations. Meta-analysis is an ideal tool to identify the genuine association while addressing between-study heterogeneity. To clarify this issue, we performed a meta-analysis of publicly independent studies. To our best knowledge, this is the first meta-analysis appraising the relationship of the selectin genes with CAD risk. As a result, we detected that carrier status for the *E-selectin* 128Arg allele was significant associated with an increase of 33% in CAD risk with remarkable heterogeneity. We hypothesized the ethnicity may be a potential origin of between-study heterogeneity. The subgroup analysis suggested that the risk increase of the Ser128Arg polymorphism on CAD among Asians were dramatically larger than Caucasians. Although the minor allele frequency of the Ser128Arg polymorphism was slightly different between Asians and Caucasians, this inconsistence can be attributed to the pleiotropic effect of the Ser128Arg polymorphism due to various genetic ancestral backgrounds. Different linkage disequilibrium patterns due to different populations were likely to be another explanation. A SNP may be in close linkage with another nearby causal variant in one ethnic population but not in another. Ethnicity might be a source of between-study heterogeneity for the Ser128Arg polymorphism. However, we were unable to evaluate the influence of ethnicity on the CAD risk of the *P-selectin* 715Pro carriers, because most of the eligible studies concerning the Thr715Pro polymorphism were conducted among Caucasians. It is difficult to estimate the modest association in other racial descents unless examining a larger population. Constructing optimized databases and replicating the results of these two polymorphisms related to CAD in different populations may benefit to draw definitive conclusions.

As well as the obvious impact of ethnicity, distinct heterogeneity can be observed when comparing different disease types (e.g., CAD versus MI). Although both CAD and MI most commonly result from atherosclerotic occlusion of the coronary arteries and share many of the same inflammatory factors, the pathological manifestations of CAD and MI are quite different. Acute atherosclerotic plaque disruption with thrombosis, which compromises coronary blood flow, is the key process of MI[3]. Although some effects on leukocyte adhesion of the E- selectin and P-selectin are overlapping, these two members of the selectin family may partly differ in function. Besides the first adhesive cellular step, E-selectin also mediates the immune response of injured endothelial cells[45]. E- selectin binding to the sialylated Lewis^x^ antigen (sLe^x^) is regarded as the preliminary step of endothelial activation, damage or turnover and serves as a molecular marker for atherosclerosis [46].Reduction in the expression of E-selectin protects against atherosclerosis in apolipoprotein E-deficient mice [47]. We identified a strong susceptibility of the Ser128Arg polymorphism to disease outcome in CAD. Substitution of E-selectin EGF domain residue 128Ser with an arginine results in an alternation in E-selectin protein ligand binding specificity and an enhanced adherence of monocyte and neutrophil to the activated endothelium in vascular areas [40], [48]. The bound and activated leukocytes and the cytokine mediators exacerbate atherosclerosis progression. By contrast, the *P-selectin* Thr715Pro polymorphism exhibited as a significant protect factor of MI but not CAD. It is could be a likely explanation if the protective effect of the 715Pro allele was directed against thrombosis rather than atherosclerosis[33]. P-selectin is localized in storage granules of platelets and the Weibel-Palade bodies of endothelial cells. P-selectin can interact with P-selectin glycoprotein ligand 1(PSGL-1) expressed on leukocytes and triggers the synthesis of pro-coagulatory tissue factor and the subsequent release of tissue factor positive microparticles from leukocytes[49]. This is a key process of atherothrombosis. The Thr715Pro polymorphism, located 15 amino acids in front of the transmembrane domain, is a non-conservative amino acid exchange. The 715Pro impairs terminal glycosylation of P-selectin and has poor sensitivity of glycan-modifying Golgi enzymes, which then lead to affect the export of P-selectin on the cell surface and inefficient adhesion compared to the wild-type [50]. The less P-selectin molecules result in the generation of fewer platelet-monocyte aggregates. This could probably explain the protective effect of the Thr715Pro polymorphism on MI. However, the true functional significance of these two polymorphisms in vivo remains unclear. Further studies assessing the selectin proteins expression and regulation are required to clarify the problem.

Our study still has several limitations. First, only two of the most common SNPs of the selectin genes were investigated. We could not rule out the possible influence of other rare variations or uncommon SNPs that have not been extensively studied. Considering the effect of a SNP on a multi-factorial disease such as CAD is relatively weak, particular haplotypes involving the Ser128Arg polymorphism or another functional mutation in linkage disequilibrium may have more significance. Another problem should be cautiously considered. There was evidence for the publication bias of the *E-selectin* Ser128Arg polymorphism in the meta-analysis. This is said to occur when the chance of the publication of a smaller study increases if it shows a stronger effect [51]. By contrast, the small negative results tend to be rejected for publication. We cannot rule out the possibility of language bias and the influence of publication timing. It is likely that some unpublished studies and “grey” literature (articles in languages other than English) have not been included. Larger and well-design studies from different language databases are required to ascertain the robustness of our results.

In summary, our meta-analysis expanded previous individual studies by supporting the evidence that the *E-selectin* Ser128Arg polymorphism was associated with an increased risk of CAD while the *P-selectin* Thr715Pro polymorphism could be a protective factor of MI. We suggested that the members of selectin family appear to play heterogeneous roles at the development of atherosclerosis and therefore be new candidate targets for diagnostic purposes and therapeutic interventions in atherosclerosis.

## Supporting Information

Checklist S1 **The PRISMA checklist for this meta-analysis.**
(DOC)
