# Correction: Heterogeneous Effect of Two Selectin Gene Polymorphisms on Coronary Artery Disease Risk: A Meta-Analysis

**DOI:** 10.1371/journal.pone.0091648

**Published:** 2014-02-28

**Authors:** The PLOS ONE Staff

A funding organization and grant are incorrectly omitted from the Funding statement. The Funding statement should read: This work was supported by the science and technology fund of Shanghai Jiao Tong University School of Medicine (11XJ21001), the new hundred talents program of Shanghai Municipal Health Bureau (XBR2013100) and the National Natural Science Foundation of China (810701773 & 81370397). The funders had no role in study design, data collection and analysis, decision to publish, or preparation of the manuscript.
